# Transcriptomic and Proteomic Profiling Revealed High Proportions of Odorant Binding and Antimicrobial Defense Proteins in Olfactory Tissues of the House Mouse

**DOI:** 10.3389/fgene.2018.00026

**Published:** 2018-02-05

**Authors:** Barbora Kuntová, Romana Stopková, Pavel Stopka

**Affiliations:** BIOCEV Group, Department of Zoology, Faculty of Science, Charles University, Prague, Czechia

**Keywords:** olfactory, lipocalin, chemical communication, immunity, MUP, OBP, antimicrobial cationic peptides, evolvability

## Abstract

Mammalian olfaction depends on chemosensory neurons of the main olfactory epithelia (MOE), and/or of the accessory olfactory epithelia in the vomeronasal organ (VNO). Thus, we have generated the VNO and MOE transcriptomes and the nasal cavity proteome of the house mouse, *Mus musculus musculus*. Both transcriptomes had low levels of sexual dimorphisms, while the soluble proteome of the nasal cavity revealed high levels of sexual dimorphism similar to that previously reported in tears and saliva. Due to low levels of sexual dimorphism in the olfactory receptors in MOE and VNO, the sex-specific sensing seems less likely to be dependent on receptor repertoires. However, olfaction may also depend on a continuous removal of background compounds from the sites of detection. Odorant binding proteins (OBPs) are thought to be involved in this process and in our study *Obp* transcripts were most expressed along other lipocalins (e.g., *Lcn13*, *Lcn14*) and antimicrobial proteins. At the level of proteome, OBPs were highly abundant with only few being sexually dimorphic. We have, however, detected the major urinary proteins MUP4 and MUP5 in males and females and the male-biased central/group-B MUPs that were thought to be abundant mainly in the urine. The exocrine gland-secreted peptides ESP1 and ESP22 were male-biased but not male-specific in the nose. For the first time, we demonstrate that the expression of nasal lipocalins correlates with antimicrobial proteins thus suggesting that their individual variation may be linked to evolvable mechanisms that regulate natural microbiota and pathogens that regularly enter the body along the ‘eyes-nose-oral cavity’ axis.

## Introduction

Chemical communication of the house mouse is mediated by the production of sex-biased major urinary proteins (MUP) from the lipocalin family, that due to their beta-barrel structure are able to protect and transport volatile pheromones in their hydrophobic pockets (Zidek et al., 1999; Timm et al., 2001; Sharrow et al., 2002, 2003). MUPs are deposited with urine marks (Jemiolo et al., 1992), and their ligands are detectable by chemosensory neuronal receptors in the main olfactory epithelia (MOE) and vomeronasal organ (VNO) (Buck and Axel, 1991; Moss et al., 1997; Buck, 2000; Leinders-Zufall et al., 2000). These receptors are differentially excitable under different pH (Cichy et al., 2015). The signal-containing secretions such as urine and saliva yield strain-specific responses at the accessory olfactory bulb (Kahan and Ben-Shaul, 2016) and specific responses by females to both saliva and vaginal secretions depending on their estrous phase (Ben-Shaul et al., 2010).These responses yield differential sensory representations in the medial amygdala (Bergan et al., 2014), and are responsible for physiological and behavioral effects in the receiver such as estrus induction and synchronization described elsewhere (Jemiolo et al., 1986, 1989; Jemiolo and Novotny, 1994; Ma et al., 1999; Novotny et al., 1999a,b; Sam et al., 2001).

The chemosensory neuronal receptors are encoded by ∼1700 genes and pseudogenes in the mouse genome (Ibarra-Soria et al., 2014, 2017). It has been shown that the olfactory transcriptomes are only minimally different between males and females in C57BL mice (Ibarra-Soria et al., 2014). Individual variation in olfactory receptors is environmentally modulated (Ibarra-Soria et al., 2017) and their expression differences between *Mus musculus musculus* and *M. m. domesticus* may allow subspecies recognition and assortative mating (Loire et al., 2017). However, other genes with inter-individual variation – mainly from the lipocalin family – highly expressed in nasal tissues (Shiao et al., 2012; Ibarra-Soria et al., 2014; Stopkova et al., 2016) – may also have roles in olfaction. For example, a group of eight lipocalins (e.g., *Obp2*, *Obp5*, *Mup4*, *Mup5*) in the study by (Ibarra-Soria et al., 2014) had similar (correlated) pattern of expression and variation such that one male showed at least a 130-fold increase in abundance. Thus, we aimed to determine the expression pattern of these highly expressed and highly variable genes in wild derived mice, *M. m. musculus*, and to provide sufficient evidence for their abundance and variation on the level of soluble proteome of the house mouse nasal mucosa. Furthermore, we used wild-derived mice which in general may have natural variation in the expression of proteins and which may then reveal particular expression dependencies that have not yet been detected in the laboratory mouse.

Genes for MUPs are organized in a cluster on the chromosome 4 (Logan et al., 2008; Mudge et al., 2008), and most of them are highly homologous in *M. m. musculus* (Thoß et al., 2015, 2016; Enk et al., 2016) while all genes for OBPs are located on the chromosome X (Stopkova et al., 2014). MUP variation is best explained by age and by various social factors such that they have higher expression levels upon social contacts or in social groups (Stopka et al., 2007; Rusu et al., 2008; Janotova and Stopka, 2011; Cunningham et al., 2013; Nelson et al., 2013; Thoß et al., 2015, 2016; Enk et al., 2016; Lee et al., 2017). Their expression is male-biased in the house mouse (Knopf et al., 1983; Sampsell and Held, 1985) while the level of sex-dimorphism is sub-species specific (Stopková et al., 2007; Hurst et al., 2017). MUPs are also known to vary throughout the estrous cycle in the urine (Janotova and Stopka, 2011) and vaginal secretions (Cerna et al., 2017) and thus, MUPs including MUP20 or ‘darcin’ are important components of female sexual signaling in *M. m. musculus* (Cerna et al., 2017). MUPs and other lipocalins (e.g., OBPs) are also present in the orofacial areas of the mouse head, namely in tears (Stopkova et al., 2017) and saliva (Stopka et al., 2016) as the products of lacrimal, nasal, salivary, lymphoid, and mucosal glands. Particularly, we have determined the expression pattern of several MUPs and OBPs in orofacial tissues and provided evidence that lacrimal glands produce high quantities of *Mup4*, *Lcn11*, *Obp5*, *Obp6*, and *Obp7* transcripts in the two house mouse subspecies *M. m. domesticus* and *M. m. musculus* (Stopkova et al., 2016).

At the protein level, females tend to produce higher quantities of OBPs in tears while males produce more exocrine gland-secreted peptides – ESPs, MUPs, and secretoglobins – SCGBs (Stopkova et al., 2017). MUPs and OBPs are also detected in saliva, though OBPs are not expressed in submandibular glands (Stopka et al., 2016), and thus it is likely that MUPs and OBPs are involved in the transport of particular ligands along the ‘eyes-nose-oral cavity’ axis. Saliva, thus, represents a complex mixture of proteins with their ligands where they may function as a cocktail-like combinatorial source of individual chemical cues that are detected directly by the receiver during social contact or may be spread on the fur during self-grooming, where their ligands may act as signals (Stopka et al., 2016). Thus, one of the aims of this study was to broaden the spectrum of proteins involved in chemical communication abundant in the nose and to detect their expression site.

The nostrils are both the primary site of odorant detection and a gate for pathogen infection and defense. Therefore, similar evolutionary forces might have shaped the evolution of multifunction proteins and their functions in systems for recognition of pathogens and chemical signals (Stopkova et al., 2014). Ligands associated with bacterial infections and those that are products of defeated bacteria during regulation of microbiota are also sensed via MOE and VNO via their microorganism-associated molecular patterns (MAMPs), and they are sensed in many places in the body including specific sets of chemosensory neurons in the mammalian nose (Bufe and Zufall, 2016). They also include the formyl peptide receptor-like proteins in VNO, which provide sensitivity to disease/inflammation-related ligands (Riviere et al., 2009) and presumably are responsible for the activation of bactericidal proteins. It has also been shown that neurons may directly control mucosal microbiota with specific peptides with amphipathic design, cationic charge and size in *Hydra* (Augustin et al., 2017) thus suggesting that this phenomenon is widespread. In the mouse mucosal tissues, bactericidal proteins or their genes (i.e., such as BPI proteins and cathelicidins) are co-expressed with lipocalins in the mouse olfactory transcriptomes (Ibarra-Soria et al., 2014), trigeminal ganglia (Manteniotis et al., 2013), tears (Stopkova et al., 2017), saliva (Stopka et al., 2016), and vaginal secretions (Cerna et al., 2017) and thus, our next aim was to detect a wider network of antimicrobial proteins in nasal tissues and to determine to which extent their inter-individual variation correlates with the inter-individual expression of lipocalins in the nasal cavity.

## Materials and Methods

### Ethical Standards

All animal procedures were carried out in strict accordance with the law of the Czechia paragraph 17 no. 246/1992 and the local ethics committee of the Faculty of Science, Charles University in Prague chaired by Dr. Stanislav Vybíral specifically approved this study in accordance with accreditation no. 27335/2013-17214 valid until 2019.

### Subjects, Housing Conditions, and Sample Collection

In this experiment, we used wild-derived *M. m. musculus* males and females with food provided ad libitum and under stable condition (i.e., 13:11 h, D:N, temperature *t* = 23°C). They come from first generation (F1) litters obtained from wild-caught mice bred in captivity. The parental wild individuals came from three different sites: Bruntál – 49.9884447N, 17.4647019E (M1, M6); Kladno – 50.1473356N, 14.1028508E (F5); Prague-Bohnice – 50.1341539N, 14.4142189E (F1, F2, F3, F4, M2, M3, M4, M5). Individuals from parental generations were trapped in human houses and garden shelters similarly as in our previous study (Stopkova et al., 2017). F1 individuals in our study had different parents and are not directly related. We used six different pairs for the transcriptomic part of the study and five pairs of individuals for the proteomic part (siblings to those used in transcriptomic part). All individuals were of similar age (∼90 days old) and weight. A total of 30 days prior to experiments all tested individuals were individually caged. The cages were placed in the same room to diminish potential stress from complete isolation. Protein samples were collected via nasal lavage with gentle pipetting by flushing in and out of the nose 10 μl of distilled water during 3 s intervals with 10 μl (white) pipette tips. This procedure was repeated three times per mouse.

### The Transcriptome

The vomeronasal organ and olfactory epithelia (i.e., mixed samples from left and right sides) were dissected and immediately placed into RLT buffer (Qiagen) and homogenized in MagNALyser (Roche) for 30 s at 6000 rpm. RNA was isolated using the RNeasy Mini Kit (Qiagen) according to the manufacturer’s protocol with on-column DNase I treatment. The purity and concentration of eluted RNA was measured with NanoDrop ND1000. The quality of RNA was checked with High Sensitivity DNA Assay on 2100 Bioanalyzer (Agilent). RNA was stored at -70°C. For the next step, we selected only high quality samples (RIN ∼8) from 6 male and 5 female individual replicates each containing the two tissues (MOE, VNO). cDNA library was prepared with TruSeq Stranded mRNA LT Sample Prep Kit (i.e., a total of 22 samples/two kits). Illumina MiSeq sequencing was performed with MiSeq^®^ Reagent Kit v3 (600 cycles) in two runs where MOE and VNO were sequenced separately. Average length of paired-end reads was always between 300 and 350 bp.

### Data Organization and Manipulation

Illumina MiSeq fastq files were used for filtering and trimming the paired end reads with Cutadapt, which finds and removes adapter sequences, primers, poly-A tails, and other types of unwanted sequence from sequencing reads. We set the minimum read length to 30 bp, trimming quality threshold was set to 30, and 10 nucleotides were removed from the 5′ ends. We used STAR (Dobin et al., 2013) for mapping individual sequences to the *Mus musculus* reference genome (GRC38). Maximum number of mismatches threshold was set to 5.0 while 0.5 was used as the lowest level for the normalization of alignment score to a read length and for the normalization of numbers of matched bases to read length. The genome mapping generated output files (^∗^.sam), which we converted to bam files, and sorted using SAMtools (Li et al., 2009). SAMtools were also used to generate ‘fasta’ tables from which we calculated N50 values (Earl et al., 2011) in R software to make sure that there is no variation in gene mapping. The number of fragments aligned to each gene was counted using the HTSeq package with the script *htseq-count* (Anders et al., 2015). HTSeq was thus used to generate the input files (i.e., Count tables) for further analyses. These tables contain Ensembl gene ids as well as the gene names, and are provided in **Supplementary Data Sheet 1**.

### Differential Expression Analysis

Differential expression was analyzed in R software using the *DEseq* routine within the *Bioconductor package* (Gentleman et al., 2004). Variation between replicates was calculated with the function *estimateDispersions*, using *per-condition* as the method. Genes were considered to be differentially expressed if they had an adjusted *p*-value of 0.05 or less [equivalent to a false discovery rate (FDR) < 5%]. To decrease potential influences of transcriptome size and sequencing depth differences on the detected differentially expressed genes, we used the size-factor vector normalization, and the variance-stabilizing algorithm, which reduces the sampling bias due to a high dispersion (variation) of counts with low expression. Thus, we obtained normalized counts, which we further subjected to the analysis of differentially expressed genes. We used normalized numbers of counts instead of FPKM values (Fragments Per Kilobase of transcript per Million mapped reads) for the fact that our statistics focused on the within-gene comparison between males and females and not the between gene comparison. However, we have also calculated FPKM (the same equation as in Ibarra-Soria et al., 2014) values and generated both plots to visualize expression patterns. When dispersion values are plotted against the means of the normalized counts, it is common that data with a low mean of normalized counts have higher levels of dispersion than high expression data due to a lower coverage of the low expression data. We used the expectation-maximization algorithm provided in the *Mixtools* Bioconductor package (Benaglia et al., 2009), using all genes with at least one fragment count per replicate, for each of the two tissues. Thus, we removed rows with zero values from the mixture of distributions, which makes the statistical fitting more robust and focused only on relevant data. We identified a mixture of two normal distributions within the *n*-binomial distribution of our data, and for statistical analysis, we only tested differences between males and females for those genes that have on average (per library) more than four reads (MOE) or five reads (VNO).

### RNA-seq Data Availability

The transcriptome data is provided as bam files in ‘Sequencing Read Archive’: www.ncbi.nlm.nih.gov/sra, SubmissionID: SUB2895984, BioProject ID: PRJNA395697.

### nLC-MS^2^ Analysis

The procedures for protein digestion were as described in Stopkova et al. (2017). We used nano-reversed-phase columns for nLC-MS^2^ analysis (EASY-Spray column, 50 cm × 75 μm ID, PepMap C18, 2 μm particles, 100 Å pore size**)**. Mobile phase buffer A was composed of water, and 0.1% formic acid while mobile phase B contained acetonitrile, and 0.1% formic acid. Samples were loaded onto a trap column (Acclaim PepMap300, C18, 5 μm, 300 Å Wide Pore, 300 μm × 5 mm, 5 Cartridges) for 4 min at 15 μl/min, loading buffer was composed of water, 2% acetonitrile and 0.1% trifluoroacetic acid. After 4 min, we switched the ventile and Mobile phase B increased from 4 to 35% B at 60 min, 75% B at 61 min, hold for 8 min, and 4% B at 70 min, hold for 15 min until the end of run. Eluting peptide cations were converted to gas-phase ions by electrospray ionization and consequently analyzed on a Thermo Orbitrap Fusion (Q-OT-qIT, Thermo) using the same parameters as in (Stopkova et al., 2017).

### Protein Analysis

We analyzed five males and five females. Each individual sample was measured twice and results averaged. LC-MS data were analyzed and quantified with MaxQuant software (version 1.5.3.8) (Cox et al., 2014). The FDR for identification of all proteins and peptides was set to 1%, and a minimum peptide length was set to 7 amino acids. We used the Andromeda search engine for the MS/MS spectra search against our modified Uniprot *Mus musculus* database (downloaded on June, 2015), containing 44900 entries. Because Uniprot contains duplicates and partial sequences, we modified our databases such that all MUP, OBP sequences were removed and instead of them we have added a complete list of MUPs from Ensembl database, and OBPs from NCBI (Stopkova et al., 2016). Next, we added those Tremble sequences that were missing in Uniprot, for example including KLKs, BPIs, SPINKs, SCGB/ABPs, and LCNs. We set the enzyme specificity as C-terminal to Arg and Lys, also allowing cleavage at proline bonds (Rodriguez et al., 2008) and a maximum of two missed cleavages. Quantifications were performed with the label-free algorithms (Cox et al., 2014) using a combination of unique and razor peptides.

All statistical analyses were performed in R software (Crawley, 2007). The dataset was normalized to diminish potential differences due to differential protein extractability and also due to potential differences caused by different signal intensities between samples. We used a normalization based upon quantiles, which normalizes a matrix of peak areas/intensities with the function normalize.quantiles from ‘preprocessCore’ routines under the Bioconductor package (Bolstad et al., 2003), which is based upon the concept of a quantile–quantile plot extended to n dimensions. To check that the data distribution conforms to the same type of distribution after normalization, we used ‘mixtools’ (Gentleman et al., 2004). Second, we used the Power Law Global Error Model (PLGEM) (Pavelka et al., 2004) to detect differentially expressed/abundant proteins using the functions plgem.fit and plgem-stn (Gentleman et al., 2004). Due to similar statistical properties between transcriptomic and proteomic data – namely the *n*-binomial distributions of signal values (i.e., deviating from normality) – it has proved to be an amenable model for the quantification of label-free MS-based proteomics data (Pavelka et al., 2008). We calculated the signal-to-noise ratio – STN [equation provided in citation (Pavelka et al., 2008)], to stabilize unequal variances by penalizing proteins that have higher variance in each class more than those proteins that have a high variance in one class and a low variance in another (Pavelka et al., 2004). The analysis of differences between males and females and the calculation of *p*-values is performed with the resampled STNs. Original LC-MS/MS data are provided in **Supplementary Data Sheet 2**, while normalized and annotated data are provided in **Supplementary Data Sheet 3**. For our multiple correlation analysis, we used Pearson correlations and the Benjamini-Hochberg *p*-adjusted values using the ‘*psych*’ routines under the Bioconductor package (Bolstad et al., 2003). Data output from correlations are provided in **Supplementary Data Sheet 4**. To detect protein–protein association networks and further information on proteins, we used STRING database which is available online at http://string-db.org/ (Szklarczyk et al., 2017).

## Results

### Transcriptome: mRNA-seq of MOE and VNO

**FIGURE 1:**
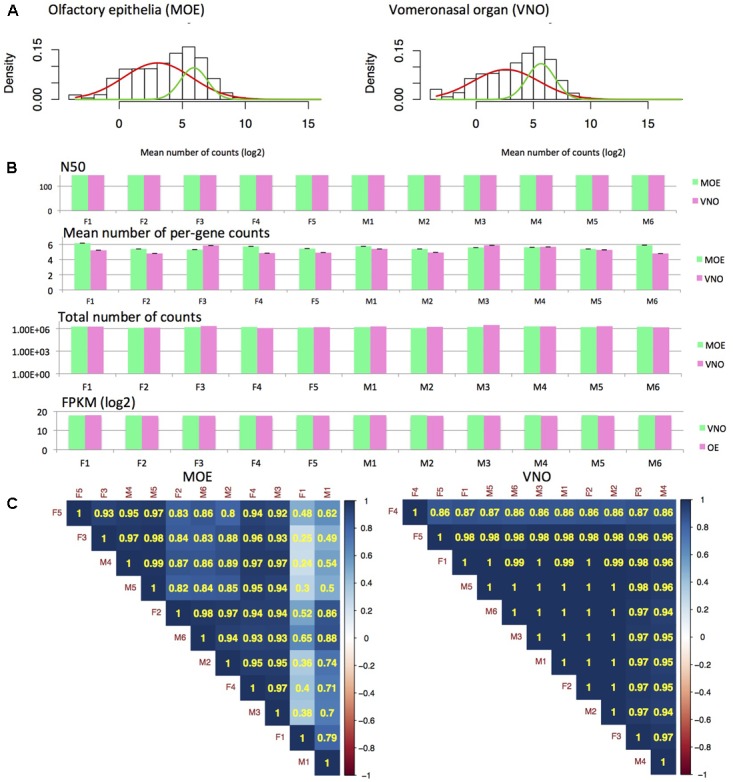
RNA-seq analysis. Fitting mixture distributions reveals the two groups of low and high expression data **(A)** from MOE and VNO. The two solid curves shown in the plots (red, green) correspond to the individual Gaussian density components in the mixture distribution, each scaled by the estimated probability of an observation being drawn from that component distribution. Analysis of read length revealed similar N50 (of reads) and sum of FPKM values for all samples and tissues **(B)**. Thus, data reduction also yielded highly similar datasets with similar mean (± SE) and total numbers of counts **(B)**. Correlation analysis using Spearman’s rank correlation (*p* < 0.001) revealed that there is a higher variation between MOE samples than VNO samples and that hierarchical clustering method does not separate males from females **(C)**.

To characterize gene expression in MOE and VNO, we extracted and sequenced whole olfactory epithelia and vomeronasal organ and generated the MOE and VNO transcriptomes. We have calculated N50 and FPKM values, which revealed that the original datasets were highly similar (N50: 175.2 ± 5.7 mean ± SD). Similarly (Ibarra-Soria et al., 2014), we have detected a mixture of two normal distributions comprising low-expression (red model-fitting curve) and high-expression (green curve) data, **Figure 1A**. Next, we obtained the posterior *p*-values with which particular data points fall onto one or another distribution within the mixture of the two normal distributions. For further analyses, we have reduced our datasets such that those points that on the level of *p* < 0.05 have fallen to a low-expression data distribution (i.e., the red fitting curve in **Figures 1A,B**) were not analyzed. This data reduction generated highly similar datasets, containing 12023 MOE transcripts and 13510 VNO transcripts, and with highly similar total numbers of counts (∼10^6^) and the mean numbers of counts, **Figure 1B**. The reduction influenced only data with low expression. For example, we have detected 5 formyl-peptide receptors (*Fpr*), 12 olfactory receptors (*Olfr*), 259 vomeronasal receptors (*Vmnr*) in VNO raw data, and after the data reduction this decreased to 2 Fprs, 1 *Olfrs*, and 125 *Vmnr*s. In MOE, we have detected 2 *Fpr*s, 6 *Taar*s, and 671 *Olfr*s, and after the reduction our data contained just 36 *Olfr*s (**Supplementary Data Sheet 1**). Next, we calculated Spearman’s rank correlations between each pair of individuals. All correlations after Benjamini-Hochberg corrections were positive and significant on the level of *p* < 0.001. However, it is obvious from **Figure 1C** that there is a higher between-sample variation in MOE than in VNO.

### Transcriptome: Differentially Expressed and Highly Expressed Genes

**FIGURE 2:**
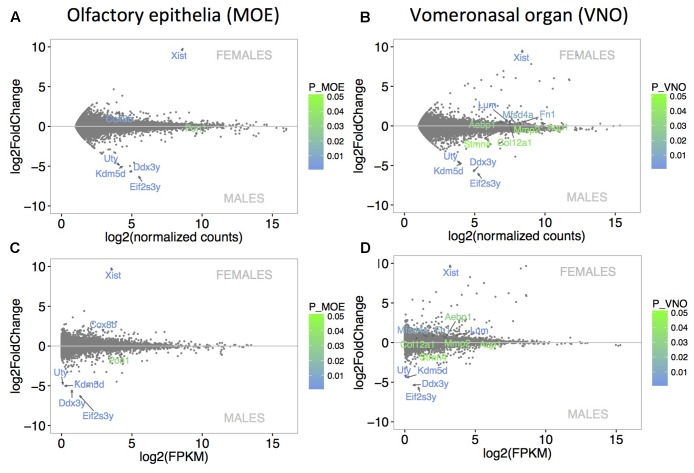
Analysis of differentially expressed genes. MA plots demonstrate that the level of sexual dimorphism is extremely low in MOE **(A)** and VNO **(B)** with only few data points being significant sexually dimorphic. Similar pattern of low sexual dimorphism is also visualized using FPKM values in **(C)** MOE and **(D)** VNO. *X*-axis represent the basal mean of normalized counts **(A,B)** or FPKM **(C,D)**, while the *Y*-axis represents particular fold differences. The level of significance (males vs. females) is scaled from green (*p* < 0.05) to blue (*p* < 0.01) and only significant points are annotated with transcript names.

The level of sexual dimorphism in the expression of MOE (**Figure 2A**) and VNO (**Figure 2B**) transcripts was extremely low. Similar pattern of expression is also demonstrated with FPKM values in **Figure 2C** (MOE) and **Figure 2D** (VNO). Only 7 out of a total of 12023 transcripts (0.06%) were sexually dimorphic in MOE (the 4 male-specific transcripts included *Eif2s3y*, *Kdm5d*, *Ddx3y*, *Uty*, and the male-biased *Pon1*, and the female-specific transcript *Xist* and the female-biased transcript *Cox8b*). A total of 13 out of 13510 expressed transcripts (0.1%) were sexually dimorphic in VNO with the male-specific *Eif2s3y*, *Ddx3y*, *Kdm5d*, *Uty*, and male-biased *Stmn4*, and with female-specific *Xist*, and female-biased *Lum*, *Fn1*, *Mfsd4a*, *Aebp1*, *Mmp2*, *Aqp1*, and *Col12a1*. They are coded by genes on sex chromosomes, and have been also detected in a previous study using higher sequencing depth (Ibarra-Soria et al., 2014). No lipocalins were detected as sexually dimorphic in this study, however, some of them were dimorphic on *p* < 0.05 (e.g., female-biased *Obp7*, *Mup5*), but when compared to other genes and using *p*-adjusted values they are no longer significant.

The distribution of highly expressed genes was different in MOE and VNO and similar values were detected using reads and FPKM (only FPKM > 1% are shown). In VNO, the first 50 genes (from 13510 genes) accounted for 50% of all fragments while in MOE the distribution is less extreme with 250 genes (from 12023 genes) accounting for 50% fragments in the original (unfiltered) dataset. Lipocalins accounted for 34% of all fragments (43.5% FPKM) in VNO including the most abundant genes (listed here in decreasing order based on the number of reads with FPKM-values > 0.5% provided in brackets) – *Lcn14* (15.7%), *Lcn13* (14.1%), *Lcn3* (12.5%), *Lcn4* (0.7%), *Obp1*, *Obp2*, *Obp5*, and *Mup4*. In MOE, lipocalins accounted for 16% of all fragments (12.9% FPKM) with most abundant genes being – *Obp2* (3.2%), *Obp1* (2.8%), *Obp5* (1.8%), *Obp8* (1.2%), *Mup4* (1.1%), *Lcn13* (1.2%), *Lcn14* (1.1%), *Lcn11* (0.76%), *Mup5* (0.54%), *Obp7*, *Obp3-p*, and *Lcn3*. Antimicrobial proteins also represented high proportions of detected genes. VNO is characterized by *Wfdc18* (1.3%), *Bpifa1* (0.94%), *Bpifb9a*, *Bpifb9b* (1% of all fragments which equals to 1.84% FPKM) while in MOE, we have detected highly abundant *Bpifb9a* (1.3%), *Bpifb9b* (1.17), *Bpifa1* (0.94%), *Bpifb3*, *Wfdc18*, *Bpifb5*, *Bpifb4*, *Bpifb6*, *Bpifb1* (11% of all fragments which equals to 4.8% FPKM). Interestingly, number of counts and FPKM values correlate in VNO and show that *Lcn14*, *Lcn13*, *Lcn3*, etc., are the most common fragments as well as the most expressed genes. In MOE, however, *Obp2*, *Obp1*, and *Obp5* are the most abundant fragments but their expression values (FPKM) drop to the fifth place (*Obp2*) with several unprocessed pseudogenes (Gm10925, Gm13340, Gm29216, Gm28437) accounting for the top 10% of the most expressed genes.

### Soluble Proteome of the Nasal Cavity

**FIGURE 3:**
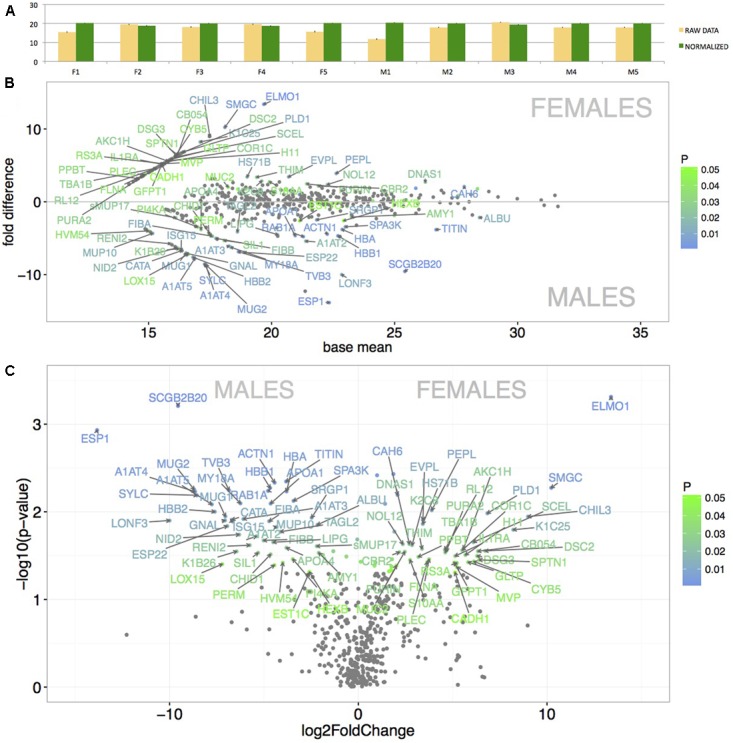
Graphical representation of the mean protein signal intensities from LC-MS/MS (*X*-axis) and of particular fold differences between males and females. Before normalization, the data revealed some variation between individuals (**A** – yellow bars). However, after the quantile-normalization procedure (**A** – green bars), the mean value and SE bars show almost no variation between the samples. Significant differentially expressed proteins are demonstrated with MA plots **(B)**. PLGEM model was involved in testing the differences in normalized signal intensities between males and females **(B)**. The level of significance (males vs. females) is scaled from green (*p* < 0.05) to blue (*p* < 0.01) and only the data points with FC > 2 are annotated with protein names. The *X*-axis represents the basal mean of signal intensities in **(B)**. The dependence of particular fold changes on *p*-values is provided using the volcano plots in **(C)**.

**FIGURE 4:**
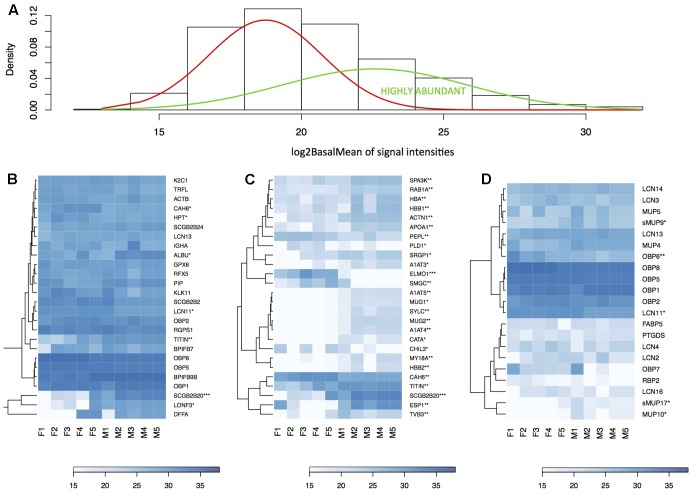
Analysis of the nasal cavity proteome. Graphical representations of protein signal distributions **(A)** reveal the two groups of high protein-abundance data (green curve) and low abundance data. Similarities between proteins and individuals were detected with a hierarchical clustering method in heatmaps using complete linkage and Euclidean distance: **(B)** the top 5% of highly expressed proteins include, e.g., LCN11, LCN13, OBP1, OBP2, OBP5, OBP8; **(B)** the top 5% of the most significant sexually dimorphic proteins (*p* < 0.01) include, e.g., ESP1 and SCGB2B20. There is a notable variation between individuals in protein abundances **(B–D)**. Note that the expression of most lipocalins is non-dimorphic with the exception of LCN11, and the group-B/central MUPs – sMUP9, MUP10, and sMUP17. ^∗^*P* ≤ 0.05, ^∗∗^*P* ≤ 0.01, ^∗∗∗^*P* ≤ 0.001).

We have generated the proteome of the nasal cavity of the house mouse, *M. m. musculus* and detected a total of 673 proteins. Next, we reduced our data such that only the proteins that were detected in three or more individuals and with median expression per row being higher than 1 were quantile-normalized and further analyzed (i.e., 517 proteins). Data normalization has resulted in highly similar datasets with similar data distribution (**Figure 3A**) thus decreasing the potential of obtaining false positive values. Our aim was to identify those proteins that are sexually dimorphic (**Figures 3B,C**) and those that within the binomial protein distribution (**Figure 4A**) represent the top 5% of the most abundant proteins (**Figure 4B**) that may characterize the proteome of the mouse nasal cavity.

Resulting mean value differences between males and females are visualized with MA plot in **Figure 3B** and in the volcano plot in **Figure 3C** (only protein names with *p* < 0.05 and fold change FC > 2 are shown). The top 5% of the most sexually dimorphic proteins are demonstrated in **Figure 4C**. The most surprising result of this study is the finding that the level of sexual dimorphism was much higher on the level of proteins than on the level of transcripts. A total of 87 out of 517 proteins (16.8%) were sexually dimorphic with 45 proteins (8.7%) being male-biased and 42 proteins (8.1%) being female-biased (*p* < 0.05). This is similar to the level of sexual dimorphism that we recently detected in the house mouse tears (Stopkova et al., 2017). When a proportional measure is used, lipocalins accounted for a total of 36.8% of the total protein quantity in males, while in females, lipocalins accounted for a total of 46.4% of the total protein quantity. Nasal OBPs are proportionally more common than MUPs (OBPs: males 33.6%, females 42.3%; MUPs: males 0.91%, females 0.43). When looking at antimicrobial proteins, BPIFB9B accounted for 19.2% of all proteins in males and 12.3% in females. When all antimicrobial proteins are counted, a total of 24.1% was detected in males and 16.6% in females. Thus, lipocalins and antimicrobial proteins accounted for the majority of proteins detected in nasal secretions of the mouse (i.e., > 50%).

### Gene/Protein Ontology of Sexually Dimorphic Proteins with STRING Database

A total of 42 proteins showed significant female bias in the nasal cavity proteome. The String database revealed significant interactions between a total of 37 proteins that were female-biased (PPI enrichment *p*-value: 0.000275). Gene ontology analysis revealed that some of those proteins (12 RS3A, RL12, PLEC, EVPL, PEPL, SCEL, DSC2, SPTN1, FLNA, COR1C, CADH1, DSG3) are involved in the structural cohesion of tissues as a part of anchoring junctions components or play roles in structural integrity of a cell (K2C8, K1C25, TBA1B). ELMO1 was the most female-biased protein and is involved in cytoskeletal rearrangements during phagocytosis (Lu et al., 2011). Second protein accounting for female-specific expression profile was SMGC (*Muc19*) which in humans is important in the homeostasis of ocular mucus (Yu et al., 2008). In mice, the expression of SMGC is restricted to mucous cells of various glandular tissues (Chen et al., 2004). MUC2 was also significantly female-biased and is known from various mucus membrane-containing organs where it forms a protective barrier against particles or excludes bacteria from the inner mucus layer. CHIL3 is a glycoprotein that plays a role in inflammation and allergy. Female-specific proteins also include enzymes such as CBR2 and AKC1H. Interestingly, CBR2 is involved in xenobiotic metabolism, while AKC1H converts progesterone to 20-alpha-dihydroprogesteron.

The most abundant as well as the most male-biased proteins in nasal mucosa were SCGB2B20 and ESP1. Expression of SCGB2B20 corroborates our previous results on the tear and saliva proteomes where their expression was also abundant and male-biased (Stopka et al., 2016, Stopkova et al., 2017). However, ESP1 (and also ESP22) transcripts were not detected either in VNO or in MOE. Interestingly, the nasal secretions also contained the male-biased group-B/central MUPs – MUP9 (FC = 4.34, *p* = 0.01), sMUP17 (FC = 2.2, *p* = 0.025) and other un-biased MUPs (e.g., MUP10) and OBPs depicted in **Figure 4D**. The analysis of male-biased genes in String databases revealed significant interactions and participations in several processes: e.g., hormone responses, responses to organonitrogen compound or complement and coagulation cascades, antimicrobial defense (e.g., PERM), detoxification (CATA), and detoxification of xenobiotics (EST1C). Many of these sex-dimorphic proteins are involved in the preventive protection from bacteria, bactericidal activity, and detoxification. Thus, we further concentrated on potentially synergistic roles of antimicrobial proteins and lipocalins in the regulation of microbiota, and which accounted for the majority of proteins and transcripts in our datasets.

### Correlations of Lipocalins with Antimicrobial Proteins (AMP)

**FIGURE 5:**
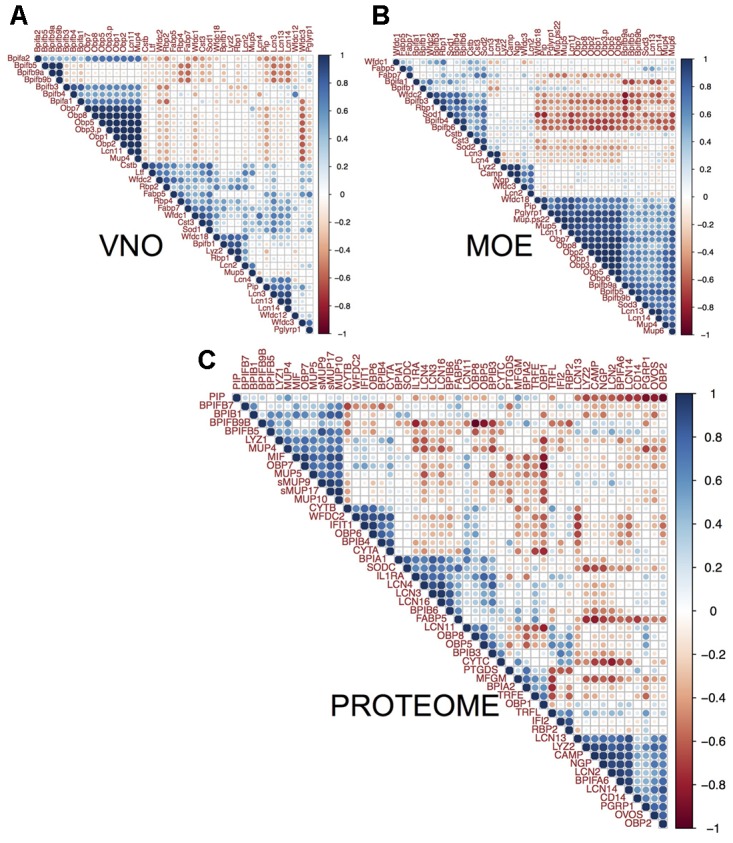
Correlation between lipocalins and antimicrobial proteins. We compared individual patterns of protein abundances. The three multiple correlation plots (correlated from –1 to 1 scaled from red to blue) – produced by hierarchical clustering with complete linkage method and Euclidean distance in **(A)** VNO, **(B)** MOE, and **(C)** the nasal cavity proteome – demonstrate that MUPs and OBPs reach the highest correlation with the levels of particular BPI proteins. Levels of *Bpifa1*, *Bpifa2*, *Bpifb3*, and *Bpifb4* significantly correlate with *Obp1*, *Obp2*, *Obp7*, *Obp8*, *Lcn11*, and *Mup4* in VNO **(A)**. Levels of *Bpifb9a*, *Bpifb9b*, and *Wfdc18* correlate with all *Obps*, *Mup4*, *Mup5*, and *Mup6* in OE **(B)**. On the level of proteome **(C)**, MUPs (i.e., central sMUP9, MUP10, sMUP17, and outlier – MUP4 and MUP5) and OBP7 are correlated with antimicrobial LYZ1, BPIB1, BPIFB5, and to some extent with BPIFB7 and BPIFB9B. OBP2, LCN13, LCN14, and the bacterial-siderophore scavenging LCN2 are correlated with BPIFA6, LYZ2, and natural antibiotics CAMP (cathelicidin) and NGP (bectenecin). LCN3, LCN4, and LCN16 are correlated with BPIA1 and BPIB6. The bigger the circle the smaller is particular *p*-value (*p* < 0.05).

Correlations at the transcriptomic level may identify groups of protein coding transcripts may have similar regulation while the proteomic level directly identifies proteins that have similar levels of abundance in the whole nasal cavity. While the transcriptomic level identifies transcripts being expressed in the same tissue, the proteomic level may identify proteins coming from multiple tissues. The most expressed AMPs in the nasal cavity were BPIFB9B and BPIFB7 (**Figure 3B**). In our data, CAMP is also highly expressed and highly correlated with NGP (*r* = 0.95, *p* = 0.027, both are on the Chromosome 9) similarly as in the vaginal secretions of the mouse (Cerna et al., 2017) and with LCN2 (*r* = 0.95, *p* = 0.04, Chromosome 2) and marginally with LYZ2 (*r* = 0.92, *p* = 0.07, Chromosome 10). The individual levels of gene expression of *Lcn2*, *Ngp*, *Camp*, *Lyz2* are correlated in MOE (*Camp* vs. *Ngp*: *r* = 0.98, *p* = 0.00005; *Camp* vs. *Lcn2*: *r* = 0.78, *p* = 0.004, *Camp* vs. *Lyz2*: *r* = 0.95, *p* = 0.007) and the same pattern is corroborated on the level of nasal proteome (**Figure 4B**). Thus, our hierarchical clustering of correlations between AMPs and lipocalins in **Figure 5** revealed this particular functional group of proteins that are already known for their capacity to kill bacteria in order to regulate microbiota or to prevent pathogens entering the body. This is why we suggest that other identified sub-clusters may also be considered as functional units within a network of antimicrobial defense.

The MOE comprises 1 large cluster (21 transcripts) and 2 smaller clusters (9 and 5 transcripts), VNO comprises 5 smaller clusters and 1 larger cluster (11 transcripts, **Figure 5**), and the nasal proteome has 7 different clusters. The most extended cluster of significant positive correlations between AMPs, lipocalins, and the bacteria-sensing Peptidoglycan recognition protein 1 (PGRP1/*Pglyrp1*, Chromosome 7) was detected in MOE (BPI, WFDC, OBPs, and MUPs encoding trasncripts), **Figure 5B**. There (MOE) was also significant negative between-cluster correlation suggesting that these systems may have opposing functions, e.g., one repressing the other. On the level of transcripts, all *Obp*s always positively correlate with *Mup4*, *Lcn11*, and several different *Bpi* in MOE and VNO. The proteome revealed a more relaxed clustering, which is probably caused by the fact that nasal mucosa contains proteins from several different expression sources. For example, LCN3, LCN4, LCN16 correlate (on the level of *p*-adjusted < 0.05) with BPIA1, BPIB6, and SODC (superoxide dismutase 3 is involved in the degradation of reactive oxygen species). WFDC proteins (i.e., ‘Whey acidic proteins four disulphide core’) were also shown to have anti-microbial properties (Scott et al., 2011). In MOE and VNO, we have detected *Wfdc1*, *Wfdc2*, *Wfdc3*, and *Wfdc18* transcripts, while on the level of proteome we have detected only WFDC2 highly correlated with BPIB4 (*r* = 0.88, *p* = 0.0008), with the two cystatins CYTA (*r* = 0.86, *p* = 0.001), CYTB (*r* = 0.65, *p* = 0.04), and with OBP6 (*r* = 0.88, *p* = 0.0007).

Nasal cavity MUPs correlate with AMPs and with other MUPs (sMUP9 vs. BPIFB5: *r* = 0.88, *p* = 0.0008, sMUP9 vs. MUP5: *r* = 0.77, *p* = 0.009; sMUP17 vs. BPIB1: *r* = 0.83, *p* = 0.003; MUP10 vs. BPIFB7: *r* = 0.66, *p* = 0.037) at the proteome level, whereas we have only detected group-A *Mup*s in MOE and VNO transcriptomes. They were, however, also correlated with genes for AMPs. For example in MOE, *Mup4* was correlated with *Bpifb9b* (*r* = 0.78, *p* = 0.004) and with *Obp*s (*Mup4* vs. any *Obp*: *r* > 0.6, *p* = 0.02–0.005). Similarly *Mup5* was significantly correlated with all *Obp*s (*r* > 0.85, *p* < 0.001). *Obp*s in MOE were highly correlated with the bacterial receptor *Pglyrp1* (*r*∼0.9, *p* < 0.0001). In VNO, the trend in correlations was slightly less obvious probably due to a lower variation between individuals but, for example, all *Obp*s correlated with *Mup4* (*r*∼0.9, *p* < 0.0002), with *Bpia1* (*r* > 0.66, *p* < 0.02), and with *Bpifb3* (*r* > 0.6, *p* < 0.05). *Obp6* as well as *Esp1*, however, were not detected in VNO and only few *Obp6* transcripts were detected in MOE. OBP6 and some group-B MUPs (e.g., MUP10) are most likely the products of other nasal glands including the nasal-associated lymphoid tissue and of lacrimal glands. Some of these proteins (e.g., ESP1, OBP6, MUP10) may be transported to nasal cavity with tears via nasolacrimal ducts. All combinations of correlation coefficients and *p*-adjusted values are provided in the **Supplementary Data Sheet 4** and visualized in **Figure 5**.

## Discussion

We have generated MOE and VNO transcriptomes and the nasal cavity proteome of the wild-derived house mice, *M. m. musculus*. Surprisingly, the level of sexual dimorphism was extremely low at the level of transcripts and high at the level of proteins. This discrepancy is most likely caused by different mRNA half lives and post-transcription machinery (Haider and Pal, 2013), by the fact the many detected proteins have different or multiple expression sites (e.g., lacrimal glands, lymphoid tissues, etc.), and because small differences in mRNAs expression may cause high differences in the expression of their proteins [e.g., demonstrated on *Mup* mRNA vs. MUP proteins in the urine (Stopková et al., 2007) or in a large study on several inbred mouse lines in different types of tissues (Ghazalpour et al., 2011)]. The level of sexual dimorphism in our transcriptomes was even lower than that reported in the study of the laboratory mice C57BL (Ibarra-Soria et al., 2014) where sex dimorphism levels were also low at the transcript level, and being mostly caused by the X- or Y-chromosome linked transcripts. At the same time, we provide evidence that several lipocalin coding transcripts (e.g., *Lcn13*, *Lcn14*, *Obp*s) belong to the most expressed genes in both tissues. We have detected a total of 19 lipocalin transcripts in VNO and 20 lipocalin transcripts in MOE. OBP coding transcripts were present in both tissues (*Obp1*, *Obp2*, *Obp5*, *Obp7*, *Obp8*) as well as *Obp3-ps* pseudogene, while *Obp6* was absent in VNO and only small numbers of counts were detected in MOE. We have also detected *Mup4*, *Mup5* in VNO and MOE and on top, MOE also expressed *Mup-ps22* and *Mup6*. MOE and VNO equally expressed *Lcn2*, *Lcn3*, *Lcn4*, *Lcn11*, *Lcn13*, and *Lcn14*, suggesting that these lipocalins may be equally important for individuals of both sex. MOE and VNO lipocalins may scavenge for harmful ligands (Grolli et al., 2006), and transport their ligands for internalization in lysozomes (Strotmann and Breer, 2011), and may remove superfluous background odorants to make the olfactory tissues continuously functional. It makes sense that it is a mixture of different lipocalins, because their beta barrels have different biochemical properties (Phelan et al., 2014; Stopkova et al., 2014, 2016) and thus, may scavenge for a wider spectrum of ligands including hydrophobic pheromones as well as harmful organic compounds such as 4-Hydroxynon-2-enal (HNE). HNE is a product of lipid peroxidation and causes chronic inflammation in mucosal tissues (Grolli et al., 2006).

The level of sexual dimorphism in nasal secretions was surprisingly high with 8.7% of proteins being male-biased and 8.1% proteins being female-biased. This is similar to the level of sexual dimorphism that we recently determined in tears with 7% of proteins being male-biased and 7% proteins being female-biased (Stopkova et al., 2017). Some proteins from nasal secretions were not expressed by genes in MOE and VNO. These may include for example OBP6, and the male-biased exocrine gland-secreted peptides ESP1 and ESP22. ESP1 is produced by lacrimal glands, secreted with tears, and when experimentally transferred to the female vomeronasal organ, it stimulates V2R-expressing vomeronasal chemosensory neurons (Kimoto et al., 2005, 2007). In wild house mice, they are male unique in tears but male-biased at the lacrimal gland transcriptome (Stopkova et al., 2017). This study shows that ESP1 along with ESP22 are also present in adult females, though in lower quantities, and they may be involved in other as yet unknown functions. ESP1 has three α-helices with two helices being negatively charged and one being positively charged. This structural amphipathy fits the description of antimicrobial peptides (Stopka et al., 2016). Thus it is possible that nasal ESPs (i.e., including ESP1 and ESP22) are involved in the host-defense against bacteria.

Most mouse mucosal tissues produce peptides and proteins that physically break bacterial membranes. They are among the most expressed proteins in this study (e.g., BPIFB9B and BPIFB7 in **Figure 3B**) and thus we identified other AMPs with ontology searches in our data and performed multiple correlations to detect potential functional associations with lipocalins. These AMPs, for example, include natural antibiotics, CAMP and NGP, with a cathelicidine domain that forms an amphipathic alpha-helix similar to other antimicrobial peptides. Functional studies demonstrated that CAMP is a potent antibiotics against Gram-negative bacteria by inhibiting the growth of a variety of bacterial strains and is expressed by neutrophils and macrophages (Gallo et al., 1997). Specific antimicrobial activity has been demonstrated for the mouse lipocalin LCN2, which is upregulated as a response to inflammation in mucosal tissues (Goetz et al., 2002; Flo et al., 2004), and which scavenges for catecholate-type siderophores that bacteria use to sequester free iron (Flo et al., 2004). Furthermore, LCN2 plays an important role in gut homeostasis because *Lcn2* knockout mice exhibited elevated levels of gut bacteria and inflammation leading to colitis and increased MUP production (Singh et al., 2016). LCN2 is equally present in male and female saliva (Stopka et al., 2016), tears (Stopkova et al., 2017) and nose in this study.

This study builds upon several previous studies which provided evidence that MUPs (Kwak et al., 2016) and OBPs (Grolli et al., 2006) bind toxic waste, and that mice recognize infected males on the basis of their odors (Zala et al., 2004, 2015). Thus, the most interesting result of this study is evidence that OBPs, MUPs, LCNs and antimicrobial proteins belong to the most abundant proteins. Interestingly, nasal secretions also contained male-biased group-B/central MUPs depicted in **Figure 4D**. Lipocalins and AMPs are correlated and different levels of correlations between particular members are revealed by hierarchical clustering method, **Figure 5**. Correlations between the genes that are clustered close to each other on particular chromosomes (e.g., *Mup* genes on the Chromosome 4, *Obp* genes on the Chromosome X, *Lcn2*, *Lcn3*, and *Lcn4* on the Chromosome 2, *Camp* and *Ngp* on the Chromosome 9) may already have a degree of modularity (Wagner and Altenberg, 1996) that may explain why their expressions are correlated. However, epistatic interactions between lipocalin and AMP genes from different clusters and chromosomes are independent of their chromosomal positions, are non-modular and their co-expression is presumably driven by different mechanisms, still being potentially evolvable (Pigliucci, 2008). Moreover, it is advantageous for an individual to have correlated levels of lipocalins and AMPs as it provides evolvability for the whole system of antimicrobial defense, where potentially harmful organic compounds, including those from bacteria, may be scavenged by lipocalins during or after antimicrobial attacks by AMPs. Within the ‘Toxic waste hypothesis,’ we claim that these compounds – lipocalin ligands – may become signals if their levels correlate with individual quality or even sex (Stopková et al., 2009; Stopkova et al., 2014). Thus, modular as well as non-modular linked systems of antimicrobial defense are evolvable onto a system of chemical communication while retaining the original antimicrobial design because their new function (i.e., chemical communication) depends on the original function. For example, BPIs are bactericidal permeability-increasing proteins (Leclair, 2003a,b) which are male-biased in the mouse saliva (Stopka et al., 2016), and may yield sex-biased compounds from defeated bacteria and from symbiotic microbiota. They may contribute to an existing pool of salivary compounds that are recognized as individual cues by which the mice immediately recognize sex and/or an individual’s health.

In addition, major olfactory epithelia rather than VNO are directly exposed to pathogens entering the body, which is presumably why the variation between individuals is higher in MOE than in VNO, **Figure 1C**. As a result, lipocalin and AMP transcripts in MOE are highly correlated with Peptidoglycan recognition protein 1 (PGRP1/*Pglyrp1*, **Figure 5B**) which activates bacterial tool-component systems (Royet et al., 2011). As most antimicrobial proteins are non-dimorphic but variable between individuals in this study, we suggest that correlations between lipocalin expression and the clustered network of antimicrobial proteins are advantageous for an individual because lipocalins – as multifunction transporters – may function as the devices that scavenge for various degradation products of regulated microbiota and defeated pathogens. They may also present these ligands as olfactory cues in saliva or elsewhere during the passage along the ‘eyes-nose-oral cavity’ axis.

## Author Contributions

PS, RS, and BK wrote the main manuscript text and collaborated on all dissections. BK helped to annotate proteomic datasets and collected the nasal samples for protein analysis. All authors have reviewed the manuscript.

## Conflict of Interest Statement

The authors declare that the research was conducted in the absence of any commercial or financial relationships that could be construed as a potential conflict of interest.

## Supplementary Material

DATA SHEET 1 Original data from mRNA-seq containing numbers of reads per individuals and with Ensembl gene IDs.

DATA SHEET 2 Original data from LC-MS/MS containing signal intensities and peptide/protein parameters and protein IDs from Uniprot.

DATA SHEET 3 Normalized and annotated data from LC-MS/MS with *p*-values, fold changes and means calculated with PLGEM model.

DATA SHEET 4 Output from multiple correlation analysis containing correlation coefficients and Benjamini-Hochberg *p*-adjusted values.
